# Clinicopathologic significance and race-specific prognostic association of MYB overexpression in ovarian cancer

**DOI:** 10.1038/s41598-021-92352-3

**Published:** 2021-06-18

**Authors:** Orlandric Miree, Sanjeev Kumar Srivastava, Mohammad Aslam Khan, Fnu Sameeta, Srijan Acharya, Harrison Ndetan, Karan Pal Singh, Kate Louise Hertweck, Santanu Dasgupta, Luciana Madeira da Silva, Rodney Paul Rocconi, James Elliot Carter, Seema Singh, Ajay Pratap Singh

**Affiliations:** 1 Department of Pathology, College of Medicine, University of South Alabama, Mobile, AL 36617 USA; 2 Cancer Biology Program, Mitchell Cancer Institute, University of South Alabama, 1660 Springhill Avenue, Mobile, AL 36604 USA; 3 Department of Epidemiology and Biostatistics, The University of Texas Health Science Center, Tyler, TX 75708 USA; 4 Fred Hutchinson Cancer Research Center, Seattle, WA 98109 USA; 5 Department of Biochemistry and Molecular Biology, College of Medicine, University of South Alabama, Mobile, AL 36688 USA; 6 Department of Gynecologic Oncology, Mitchell Cancer Institute, University of South Alabama, Mobile, AL 36604 USA

**Keywords:** Cancer, Biomarkers

## Abstract

Late diagnosis, unreliable prognostic assessment, and poorly-guided therapeutic planning result in dismal survival of ovarian cancer (OC) patients. Therefore, identifying novel functional biomarker(s) is highly desired for improved clinical management. MYB is an oncogenic transcription factor with emerging functional significance in OC. Here we examined its clinicopathologic significance by immunohistochemistry and TCGA/GTex data analyses. Aberrant MYB expression was detected in 94% of OC cases (n = 373), but not in the normal ovarian tissues (n = 23). MYB was overexpressed in all major epithelial OC histological subtypes exhibiting the highest incidence (~ 97%) and overall expression in serous and mucinous carcinomas. MYB expression correlated positively with tumor grades and stages. Moreover, MYB exhibited race-specific prognostic association. Moderate-to-high MYB levels were significantly associated with both poor overall- (p = 0.02) and progression-free (p = 0.02) survival in African American (AA), but not in the Caucasian American (CA) patients. Consistent with immunohistochemistry data, we observed significantly higher MYB transcripts in OC cases (n = 426) than normal ovary (n = 88). MYB transcripts were significantly higher in all epithelial OC subtypes, compared to normal, and its greater levels predicted poor survival in AA OC, but not CA OC, patients. Thus, MYB appears to be a useful clinical biomarker for prognostication, especially in AA patients.

## Introduction

Ovarian cancer (OC) is the fifth most common cancer among women in the United States^1^. It is also the second most common gynecological malignancy in American women, causing more deaths than any other cancer of the female reproductive system^2^. American Cancer Society estimates that 21,410 new OC cases will be diagnosed in 2021, and it will claim approximately 13,770 lives this year^1^. It is also reported that OC disproportionately affects women of certain racial and ethnic backgrounds, especially African American (AA) women. AA women experience higher mortality than Caucasian American (CA) women regardless of tumor stage, grade, and histology despite having slightly lower incidence (9.5 per 100,000 versus 12.9 per 100,000)^3–5^. While the five-year survival rate has improved for CA women in the past decade, it has worsened in AA women with an ovarian cancer diagnosis^5^. Underlying causes of such disparity are unclear, but a late diagnosis and the lack of therapeutic options for the advanced disease are considered prime reasons for the overall grim prognosis of OC.

OC is a highly heterogeneous group of diseases, of which most (~ 90%) are of epithelial origin. The epithelial OC (EOC) is subdivided into four major histological subtypes: serous (high- and low-grade), endometrioid, clear cell, and mucinous. Serous carcinoma (SC) is the most common histological subtype (~ 70%), followed by endometrioid (EC), mucinous (MC), and clear cell carcinoma (CCC)^6–9^. Increasing evidence suggests that these EOC histotypes have different cells of origin and also vary in genetic susceptibilities and clinical responses to therapy^8,10^. Significant molecular differences also exist among histological subtypes, which remain to be characterized fully for clinical exploitation in biomarker and therapeutic development.

*MYB* is a proto-oncogene that encodes for a transcription factor protein^11,12^. Defects in the chromosomal region harboring *MYB *were first reported in human acute myelogenous leukemia^12^. Since then, its overexpression and other abnormalities have been detected in several other malignancies^13–17^. MYB plays an essential role in maintaining the undifferentiated proliferative state of immature hematopoietic cells, and its knockout causes the loss of blood cell lineages in mice and is embryonically lethal^18,19^. Information on MYB functions in OC pathobiology has begun to emerge recently^20,21^, underscoring the need to study its expression pattern and clinicopathologic significance in OC.

In the present study, we investigated MYB expression in situ by immunohistochemistry in normal and malignant ovarian tumors. We also evaluated MYB mRNA expression in OC and its clinicopathologic correlation using publicly available transcriptomic databases. Our findings demonstrate significant MYB overexpression in all EOC histological subtypes, with the most pronounced expression detected in SC and MC. MYB at the protein level correlated positively with increasing tumor-grade and clinical stages. Moderate-to-high MYB protein levels also predicted worse progression-free survival. Although no significant difference in MYB expression was observed between AA and CA OC, high MYB expression was a stronger predictor of poor overall and progression-free survival in AA women. Thus, our study suggests that MYB can be a useful diagnostic and prognostic biomarker for ovarian malignancy, particularly in AA patients.

## Results

### MYB is overexpressed in ovarian cancer

**Figure 1:**
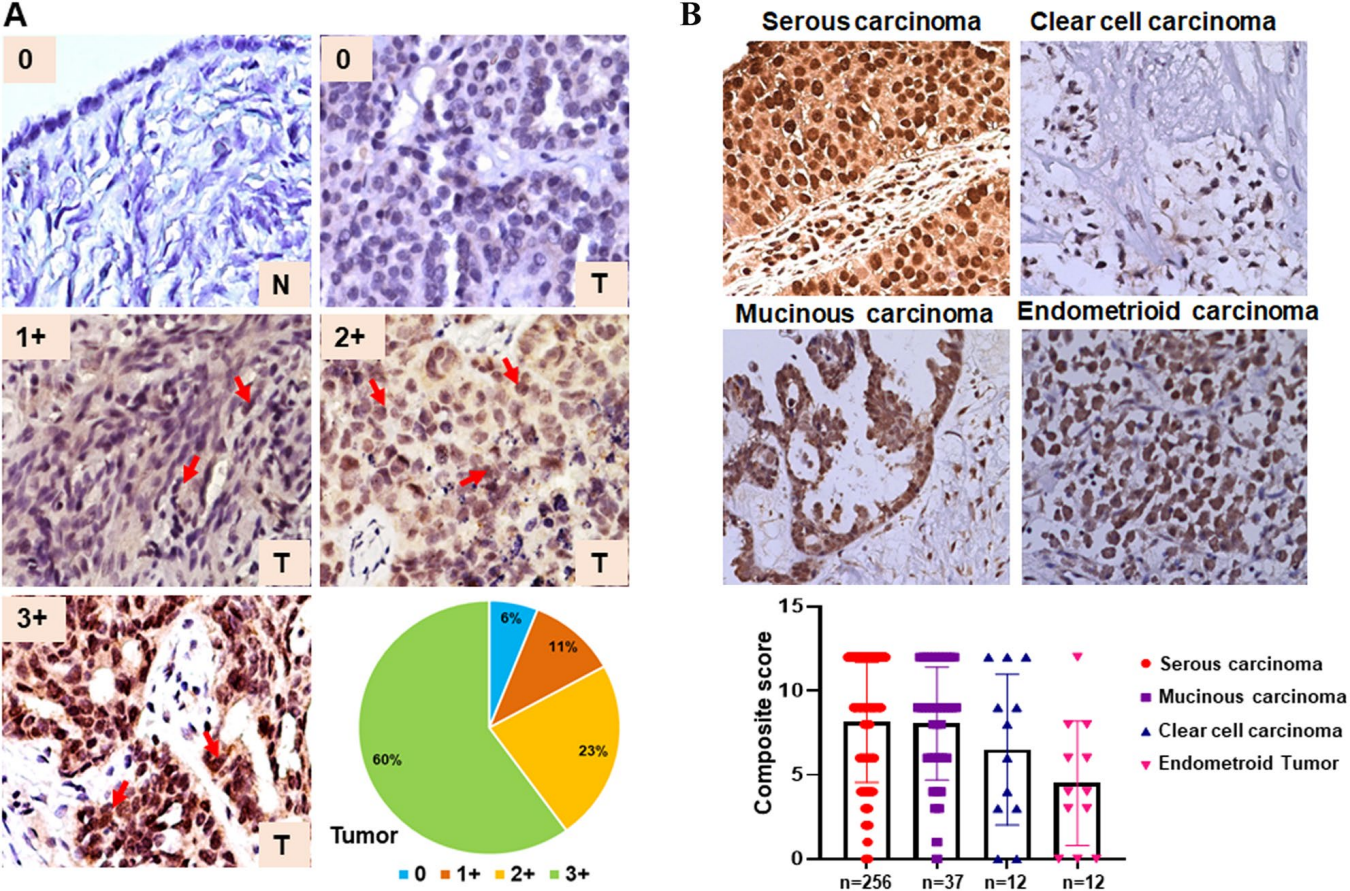
MYB protein expression in the cohort of ovarian cancer. (**A**) MYB expression in normal (N) ovary (n = 23) and tumor (T) samples (n = 373) was determined by performing immunohistochemistry. MYB staining was predominantly observed in the nucleus (arrow) with diffuse staining in the cytoplasm. Overall staining intensity ranged from 0- to 3 + in OC cases, whereas none of the normal ovary tissue sections exhibited a positive MYB staining. (**B**) A variable expression of MYB was observed in different histological subtypes of ovarian cancer (serous carcinoma, SC; mucinous carcinoma, MC; clear cell carcinoma, CCC and endometrioid carcinoma, EC). The bar diagram shows the distribution of composite score (nuclear MYB staining intensity and percent positivity) in histological subtypes of epithelial ovarian cancer. Magnification ×200.

To analyze MYB expression in OC, we performed immunohistochemical analysis on tissue sections from the normal ovary (n = 23) and ovarian tumors (n = 373). As expected, we observed predominant nuclear staining for MYB with some diffuse staining in the cytoplasm. No expression of MYB was detected in any of the normal ovarian tissues examined, whereas 94% of the OC cases stained positive for MYB. Of the positively stained OC cases, 60% (225/373) exhibited strong (3+) staining, whereas moderate (2+) and weak (1+) staining was detected in 23% (85/373) and 11% (40/373) cases, respectively (Fig. 1A). The overall mean composite score, reflective of percent positivity and staining intensity, was 7.7 ± 3.7 for all OC cases combined. Among the different histological subtypes, the highest incidence of MYB positivity was recorded in serous carcinoma (97%, 249/256) and mucinous carcinoma (97%, 36/37), followed by clear-cell carcinoma (83%, 10/12), and endometrioid carcinoma (75%, 9/12). The composite score distribution in these subtypes revealed the highest mean composite score for serous carcinoma (8.12 ± 3.6) followed by mucinous (8.05 ± 3.3), clear cell (6.5 ± 4.4), and endometrioid carcinoma (4.5 ± 3.7) (Fig. 1B). We also examined if the MYB expression varied between OC cases from CA and AA patients but did not observe any significant difference (Supplementary Figure S1).

### MYB exhibited a positive association with progressive clinical stages and histological grades

**Figure 2:**
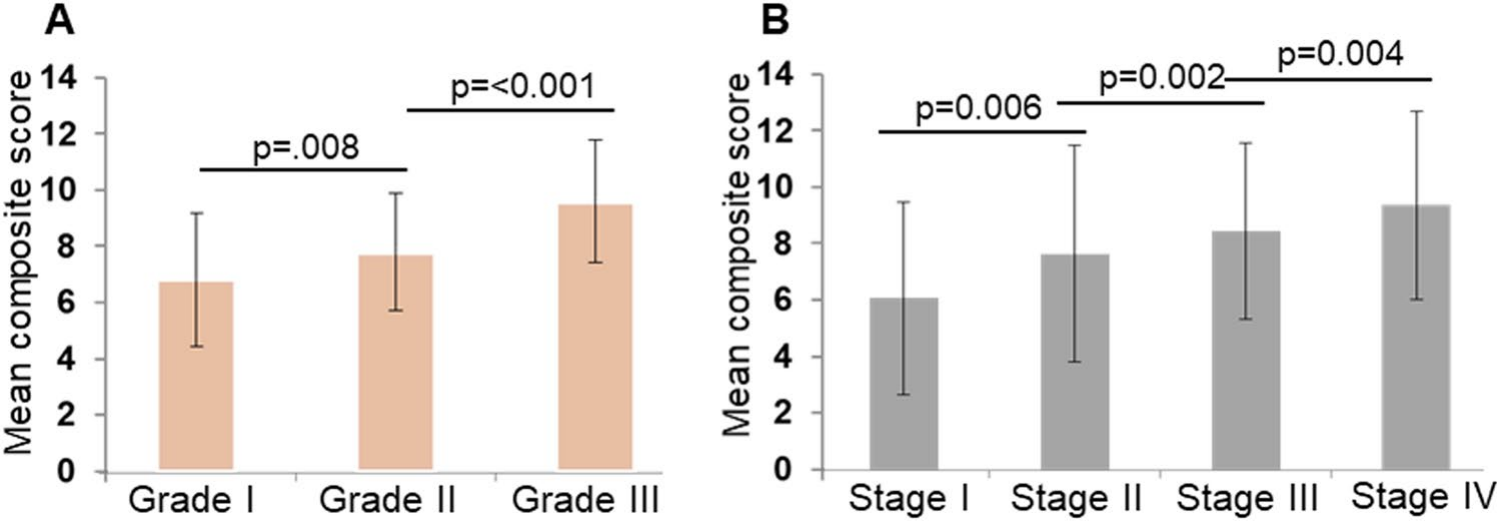
MYB expression in OC tissues of different histological grades and clinical stages. MYB protein expression levels (composite scores) in OC cases belonging to histological grades I, II, and III (**A**) and clinical stages I, II, III, and IV (**B**) were used to establish a statistically significant association. A p value of < 0.05 was considered significant.

We next examined changes in MYB expression pattern in progressive histological grades and clinical stages. Our cohort had 14% (52/373) tumors from grade I, 28% (106/373) from grade II, and 35% (131/373) from grade III. Similarly, 47% (174/373) tumors were from stage I, 11% (43/373) from stage II, 18% (69/373) from stage III, and 8% (29/373) from stage IV. Overall, we observed a positive association of MYB expression (mean composite score) with the increasing histological grade of ovarian tumors. Specifically, an increase in MYB expression significantly correlated with the grade, and it changes from grade I to II (OR = 2.579, 95% CI = 1.278–5.207, p = 0.008) and from II to III (OR = 3.751, 95% CI 2.165–6.499, p = <0.001) (Fig. 2A). Furthermore, the stage-wise progression was also found to be positively associated with increased MYB expression. While the cancer stages change from stage I to II (OR = 3.230, 95% CI 1.393–7.492, p = 0.006), II to III (OR = 5.255, 95% CI 1.866–14.800, p = 0.002), and III to IV (OR = 2.521, 95% CI 1.013–6.275, p = 0.047), respectively (Fig. 2B).

### MYB overexpression is a predictor of poor disease outcomes, especially in African American ovarian cancer patients

**Figure 3:**
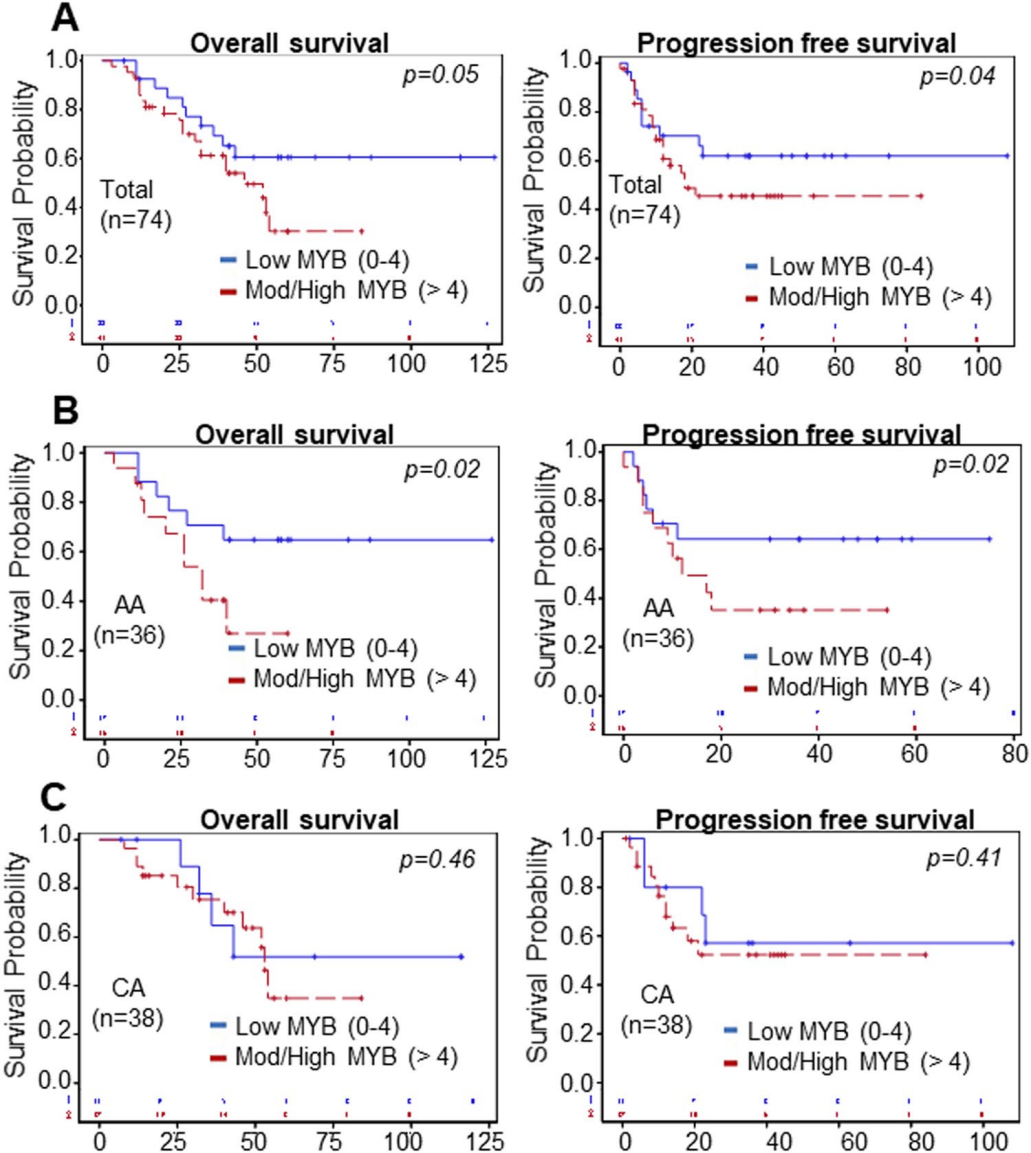
Association between MYB expression and the survival of ovarian cancer patients. (**A**) Kaplan–Meier survival analysis for the association of MYB with the overall survival (left panel) and progression-free survival (right panel) of ovarian cancer patients (n = 74). A significant association (p = 0.04) of MYB was recorded with poor progression-free survival in ovarian cancer patients when compared between low (≤ 4) versus moderate to high (˃4) MYB expressing patients. A clear trend of MYB association with overall survival was also recorded (p = 0.05). (**B**, **C**) Association between MYB expression and the survival of OC patients with AA and CA racial background. AA women with OC showed poor overall (p = 0.02 vs. p = 0.46) and progression-free survival (p = 0.02 vs. p = 0.41) than the patients of CA origin.

Since MYB expression exhibited a positive association with increasing tumor grade and stage, we next examined if MYB levels correlated with the survival of OC patients by performing Kaplan–Meier analysis. Survival data were available for a total of 74 OC cases, of which 36 and 38 belonged to AA and CA women with OC, respectively. We first used a mean composite score cut-off value of ≤ 6 as low MYB and ˃6 as high MYB to examine a survival association. Our data demonstrated a trend of poor -overall and -progression-free survival in AA OC patients with high MYB expression; however, it failed to reach the statistical significance (Supplementary Figure S2). Next, we examined the survival association by keeping the mean composite score of ≤ 4 as a cut-off for the low MYB-expressing group, and cases with a composite score of > 4 were categorized as moderate to high MYB-expressing group. Based on this criterion, we observed a significantly worse progression-free survival (p = 0.04) of the OC patients with moderate/high MYB expression than those with low MYB expression. In addition, a clear trend towards poor overall survival of the OC patients with moderate to high MYB expression was also recorded compared to the low MYB group (p = 0.05) (Fig. 3A). Since women of AA racial background have greater mortality from OC despite having a lower incidence than the CA women^22^, we examined the prognostic association of MYB separately in these groups. We observed a significantly worst overall (p = 0.02) and progression-free (p = 0.02) survival of the AA subjects with moderate to high MYB expression when compared to the low MYB expressing group (Fig. 3B). However, we did not observe a significant change in overall and progression-free survival of the CA subjects with mod/high MYB expression compared to the low MYB expressing group (Fig. 3C). Thus, our data suggest that MYB could be a stronger predictor of survival in AA women with OC diagnosis.

### Expression pattern of MYB transcript and its prognostic association in OC patients

**Figure 4:**
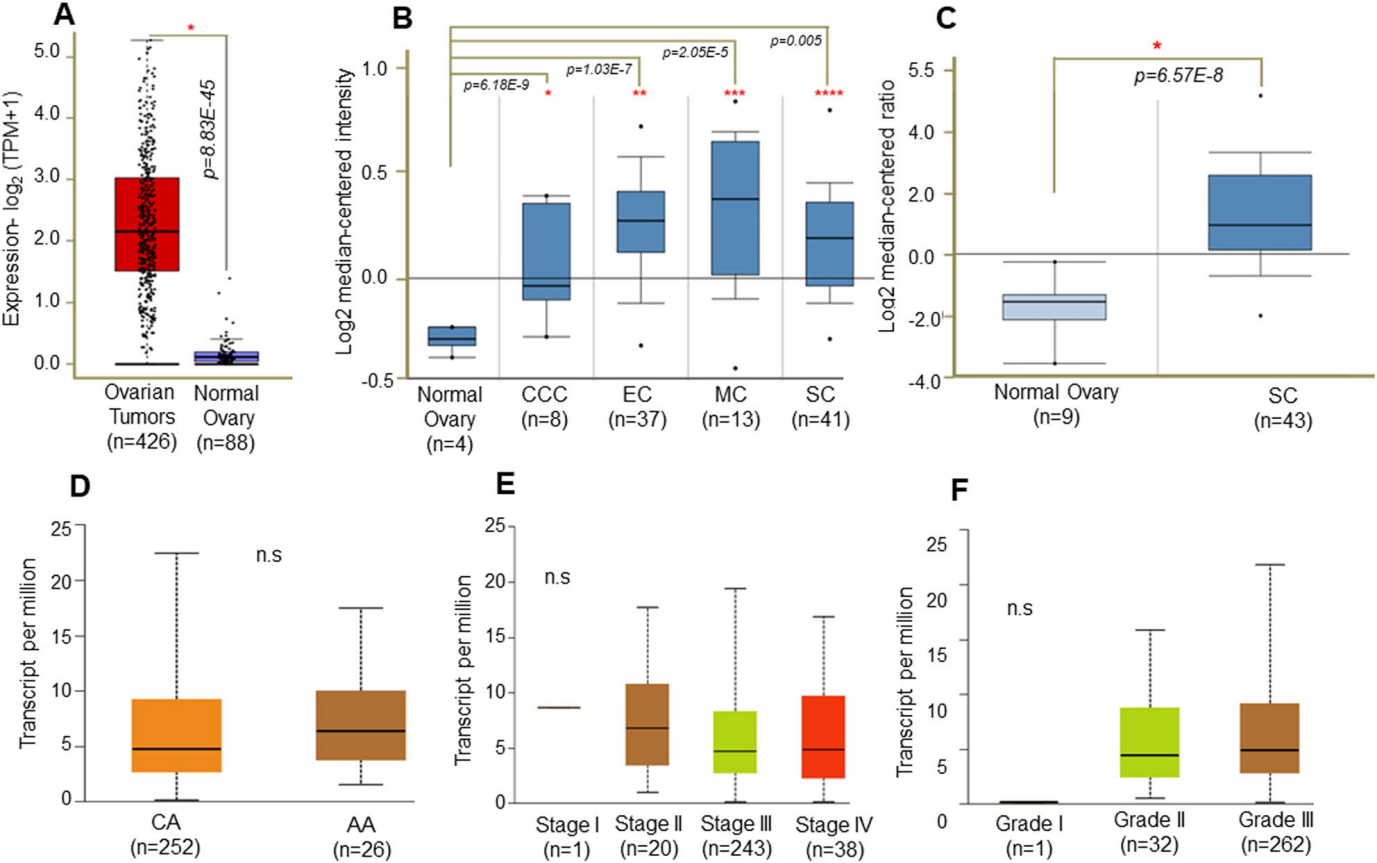
Transcriptomic analysis of MYB in ovarian cancer patients. (**A**) Significantly higher MYB mRNAs were present in ovarian cancer cases than in the normal ovary. (**B**, **C**) A variable but increased expression of MYB transcripts was also observed in different histological subtypes. (**D**) MYB transcript levels were not significantly different in AA (n = 26) and CA (n = 252) OC cases. (**E**, **F**) Similarly, no significant association of MYB mRNA levels was observed with clinical stages and histological grades. A *p* value of < 0.05 was considered significant.

**Figure 5:**
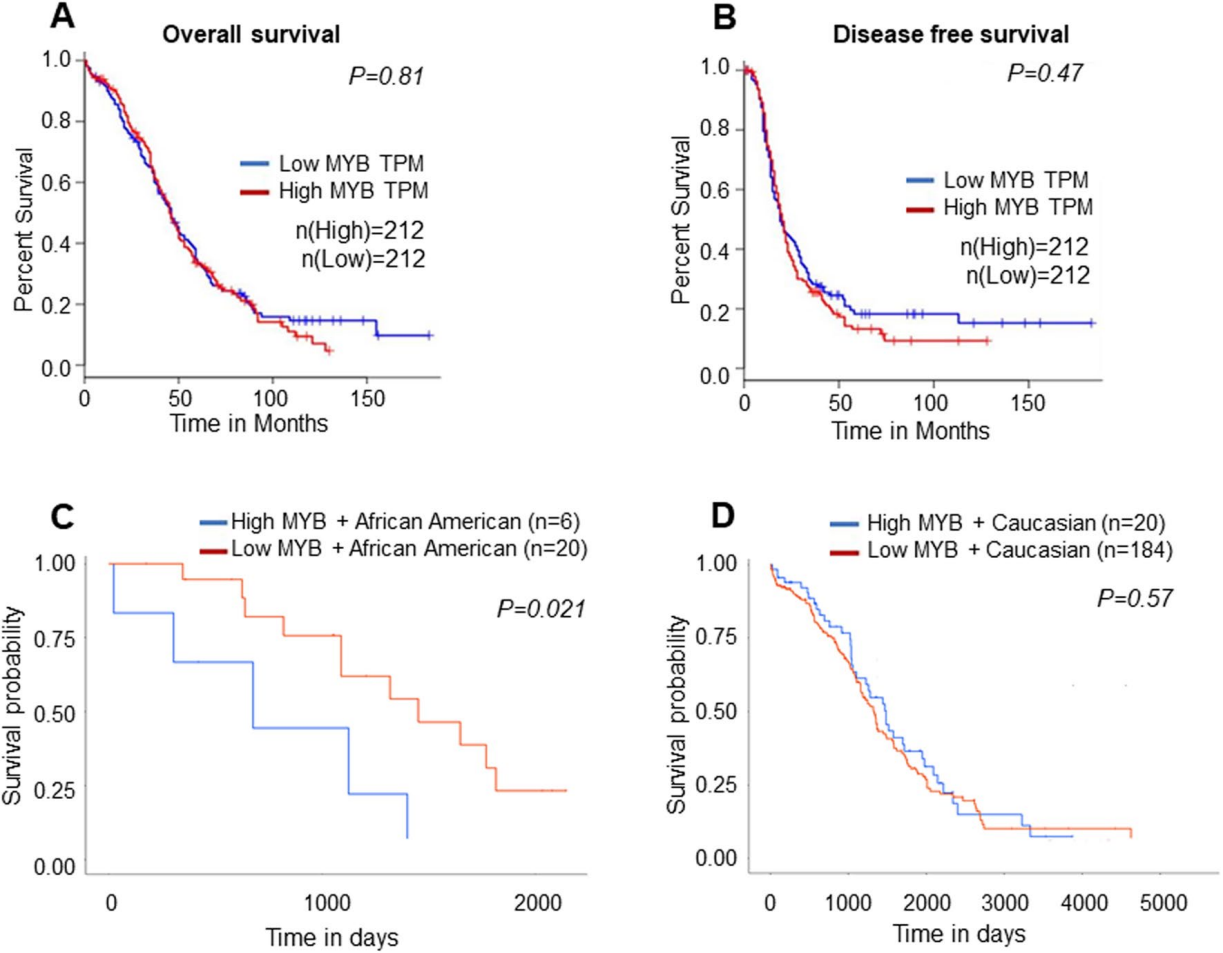
Prognostic potential of MYB transcript in overall and racially disparate ovarian cancer patients. (**A**, **B**) MYB at the transcript level did not exhibit a significant association with either overall- or disease-free survival. (**C**, **D**), African American women (n = 26) with high-MYB expression demonstrating poor disease outcome (p = 0.021), but not the Caucasian American (n = 204) (p = 0.57).

To further generate support for our observations, we next analyzed the transcriptomic data available in multiple public databases, including GTEx (Genotype-Tissue Expression), TCGA-ovarian cancer project (The Cancer Genome Atlas), and individual laboratory studies. These databases were searched by using interactive analytical platforms, GEPIA, UALCAN, and ONCOMINE. The data mining revealed significantly higher expression of MYB transcript (false discovery rate-adjusted) in a large ovarian cancer cohort (n = 426) compared to the normal ovarian tissues (n = 88) (Fig. 4A). A differential expression of MYB transcripts among histological OC subtypes was also recorded (Fig. 4B, C). No significant differences in MYB expression were recorded in AA and CA OC cases in the TCGA database (Fig. 4D). Similarly, no significant association of MYB transcript levels with clinical stages or histological grade was observed (Fig. 4E, F). MYB also did not significantly associate with overall or progression-free survival (Fig. 5A, B). When examined in AA and CA groups, a significant association of high MYB with lower survival was recorded in AA women with OC (p = 0.021) but not in CA women (p = 0.57) (Fig. 5C, D). These data suggest that MYB is aberrantly expressed at both RNA and protein levels in OC and could serve as a predictor of survival, especially in AA women with OC. Further, MYB should also be explored as a functional target associated with racially disparate clinical outcomes in AA women.

## Discussion

The scarcity of reliable screening tools for early‐stage diagnosis is one of the major challenges associated with the poor survival of OC patients^23,24^. Further, lack of clarity about the cell of origin and molecular and histological complexities make the therapeutic planning and prognostication of epithelial OC clinically challenging^6,10^. Identification of functional biomarkers associating with disease aggressiveness and clinical outcomes could allow for better therapeutic planning, disease monitoring, and the development of novel targeted therapeutics. Indeed, existing and emerging studies continue to demonstrate the strength of molecular markers in accurate histopathological and clinical diagnosis, prognosis, and treatment guidance.

The present study identified MYB as a potentially useful clinical biomarker for OC. Like other transcription factors, activated MYB is localized in the nucleus, where it regulates the expression of genes associated with different cellular processes^11,17,25^. The earliest documentation of aberrant MYB expression in OC came from a study that examined MYB transcripts in OC cell lines and a few clinical cases^26^. Our study examined MYB expression in a larger cohort of normal and ovarian cancer cases and found its widespread overexpression in all EOC histological subtypes. Staining intensity varied (weak to strong) between and within cases suggestive of the heterogeneous disease or the cells in various progression stages. The variable expression of MYB in OC cases could also represent molecular heterogeneity resulting from genetic differences or epigenetic alterations in cell populations due to their exposure to the differing tumor microenvironment.

MYB expression was not detected in any of the normal ovary cases suggesting that its dysregulation occurs following malignant transformation, and thus it could be of potential diagnostic significance. As expected, MYB expression was predominantly nuclear, although diffuse staining in the cytoplasm was also detected. Therefore, it will be interesting to pursue if MYB also has any non-nuclear functions besides gene regulation. Further, among all histological subtypes, SC and MC exhibited the highest expression. *p53* mutations have been reported in both these subtypes^10^. Thus, molecular crosstalk between MYB and p53 can aid SC and MC, requiring further validation in preclinical and clinical settings.

Irrespective of the histological subtypes, MYB protein expression positively correlated with OC's progressive histological grades and clinical stages. This is in line with an established role of MYB in cellular differentiation and promotion of malignant behavioral features^12,16,17,25,27^. It also underscores the need to delineate the molecular mechanisms involved in MYB dysregulation in OC to develop disease prevention and therapy strategies. Prediction of worst progression-free survival by the moderate-to-high MYB protein expression in OC cases further strengthens the notion that MYB plays an important role in disease aggressiveness. Moreover, stronger MYB association with poor survival in AA patients is also exciting and warrants further investigations. This is quite interesting and suggests that MYB likely acts in concert with other molecular factors in AA OC to impact tumor biology leading to racially disparate clinical outcomes. In fact, we have previously observed that increased expression of indoleamine 2.3-dioxygenase in AA OC patients associated with poor survival^28^. Thus, it will be interesting to examine a mechanistic link of MYB with other proteins exhibiting racially disparate expression in OC. It is also interesting to note that a lower cutoff of MYB expression levels provided a better correlation with survival outcomes. It suggests that MYB being a transcription factor can significantly impact the transcriptome and, as a result, cellular phenotypes upon slight increases in its expression.

Aberrant expression of MYB was also apparent at the mRNA level, suggesting that its dysregulation in OC likely occurs at the genomic (gene amplification), transcriptional and/or post-transcriptional levels. Indeed, in other cancers, both gene amplification and transcriptional upregulation of MYB have been reported^11,14^. Further, post-transcriptional regulation via microRNAs is also documented^21,29^. Unlike protein, the MYB transcript did not significantly associate with histological grades and clinical stages due to the smaller sample size. A statistically significant association of higher MYB mRNA levels with poor survival outcomes in AA patients further supports its role in racial disparity requiring further investigations.

In conclusion, our study establishes the clinical significance of MYB in OC that should be explored further in combination with other biomarkers. Functional and in-depth mechanistic studies including identification of transcriptional targets of MYB in OC should also be pursued. These efforts will yield information that could be exploited to develop MYB-targeted therapeutic strategies and clinical utilization of MYB as a useful biomarker of diagnostic and prognostic significance.

## Materials and methods

### Tissue samples, antibodies and reagents

A total of 373 ovarian cancer and 23 normal ovary tissues were used in this study. The de-identified formalin-fixed paraffin-embedded tissue sections (5 μm) were either obtained through the commercial source (US Biomax, Inc., Rockville, MD, USA) or the University of South Alabama Health Biobank under an Institutional Review Board-approved protocol. Polyclonal anti-MYB rabbit antibody was obtained from Abcam (Cambridge, MA). Betazoid DAB Chromogen Kit, Da Vinci Green diluent, peroxidazed 1, MACH 3 Rabbit HRP Polymer Detection, Background Sniper, Diva Decloaker, wash buffer (Tris-buffered saline with Tween 20), Tacha’s bluing solution (Biocare Medical, Concord, CA), ethanol (Thermo Fisher Scientific, Waltham, MA).

### Immunohistochemistry

IHC was performed on formalin-fixed, paraffin-embedded normal or ovarian tumor tissue sections as previously described^25^. In brief, tissue sections were deparaffinized, hydrated, and subjected to antigen retrieval in Decloaking Chamber (Biocare Medical, Concord, CA). After that, peroxide quenching was done with 3% H_2_O_2_ in methanol at room temperature for 30 min followed by noise blocking for 10 min with Background Sniper. Sections were then incubated with anti-MYB antibody overnight at 4 °C, washed with Tris-containing buffer, and incubated at room temperature with respective polymer and probe for 10 min each. Immunoreactivity was visualized by treating the sections with DAB chromogen followed by hematoxylin counterstaining.

### Quantification of MYB staining

All the slides were viewed under a Zeiss Axioskop 40 (White Plains, NY) and scored for the nuclear staining of MYB on a three-point scale, 1+ (weak); 2+ (moderate); and 3+ (strong), and the percent positivity on a four-point scale, 0–25% (‘1’), 26–50% (‘2’), 51–75% (‘3’), and 76–100% (‘4’) using standard scoring recommendation^30^. Weak (1+) staining in less than 10% of cells was considered negligible and given a value of ‘0’. A composite score was calculated for each section by multiplying the staining intensity and percent positivity values, ranging from 0 to 12^30^.

### In silico analysis for differential MYB transcript estimation

To examine the differential expression of MYB at the transcript level in normal ovary versus ovarian cancer, using different public databases, including GTEx (Genotype-Tissue Expression), TCGA-ovarian cancer project (The Cancer Genome Atlas), and individual laboratory studies. These databases were searched by using interactive analytical platforms, GEPIA (http://gepia.cancer-pku.cn/), UALCAN (http://ualcan.path.uab.edu/), and ONCOMINE (https://www.oncomine.org). Besides, an association of MYB mRNA with race, clinical stages, histological grades, and patient survival was also studied.

### Statistical analysis

We employed Wilcoxon, Mann–Whitney, Anova, Chi-square, Fisher’s exact tests, and logistic regression model as appropriate for all statistical data comparisons between various experimental groups. Overall survival curves were developed using the Kaplan–Meier method and compared using the stratified log-rank test. All p-values were two-sided, and all confidence intervals were at the 95% level. A *p*-value of < 0.05 was considered statistically significant. Computation for all the analyses was performed using the Statistical Analysis System (SAS).

## Supplementary Information

Supplementary Figures.

## Data Availability

The data presented in this article is available for sharing upon a reasonable request.
