# Supplementary material for: Clinicopathologic significance and race-specific prognostic association of MYB overexpression in ovarian cancer
Source: Sci Rep. 2021 Jun 18;11:12901. doi: 10.1038/s41598-021-92352-3 (PMC8213794; doi:10.1038/s41598-021-92352-3)
Supplement: Supplementary file 1 — Supplementary Figures. [file 41598_2021_92352_MOESM1_ESM.docx]

**Clinicopathologic significance and race-specific prognostic association of MYB overexpression in ovarian cancer**

Orlandric Miree^1,2^, Sanjeev Kumar Srivastava^1,2^, Mohammad Aslam Khan^1,2^, Fnu Sameeta^1^, Srijan Acharya^1,2^, Harrison Ndetan^3^, Karan Pal Singh^3^, Kate L Hertweck^4^, Santanu Dasgupta^1,2,5^, Luciana Madeira da Silva^6^, Rodney Paul Rocconi^6^, James Elliot Carter^1^, Seema Singh^1,2,5^, Ajay Pratap Singh^1,2,5,*^

Composite score

*p=0.508*

AA

CA

**Supplementary Figure 1.** MYB expression in AA (n=36) and CA (n=38) OC samples. The bar graph shows the distribution of composite score in epithelial ovarian cancer. p value of < 0.05 was considered as significant.


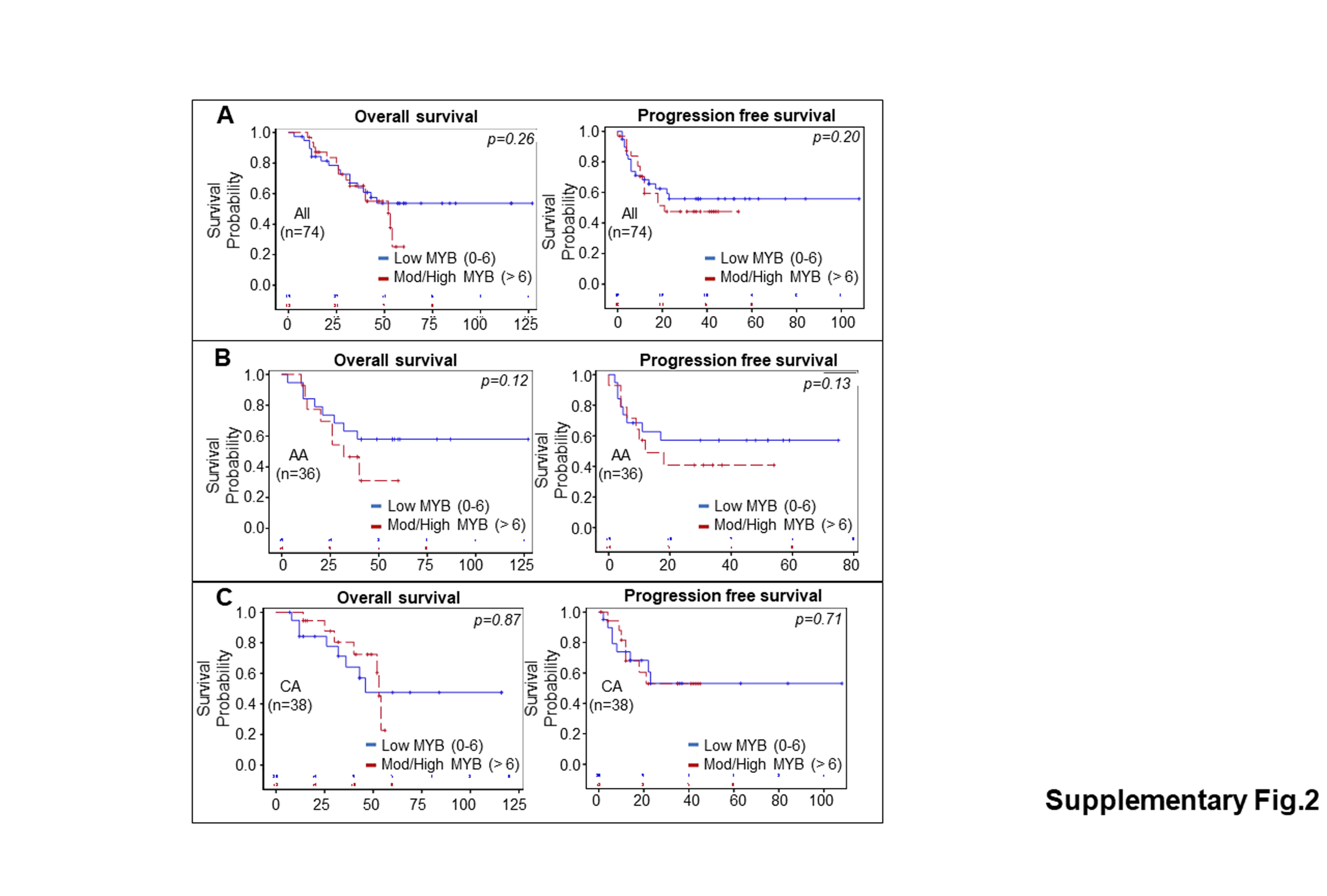


**Supplementary Figure 2.** **Association between MYB expression and ovarian cancer patients’ survival.** (**A-C**) Kaplan-Meier survival analysis for the association of MYB with the overall and progression-free survival of all as well as AA and CA ovarian cancer patients with a cut-off value, low (≤6) versus moderate to high (˃6) MYB. A p value of < 0.05 considered as significant.
